# Human intrahepatic CD69 + CD8+ T cells have a tissue resident memory T cell phenotype with reduced cytolytic capacity

**DOI:** 10.1038/s41598-017-06352-3

**Published:** 2017-07-21

**Authors:** Femke Stelma, Annikki de Niet, Marjan J. Sinnige, Karel A. van Dort, Klaas P. J. M. van Gisbergen, Joanne Verheij, Ester M. M. van Leeuwen, Neeltje A. Kootstra, Hendrik W. Reesink

**Affiliations:** 1 Department of Gastroenterology and Hepatology, Academic Medical Center, Amsterdam, The Netherlands; 2 Department of Experimental Immunology, Academic Medical Center, Amsterdam, The Netherlands; 3 Department of Hematopoiesis, Sanquin Research and Landsteiner Laboratory, Academic Medical Center, Amsterdam, Netherlands; 4 Department of Pathology, Academic Medical Center, Amsterdam, The Netherlands

## Abstract

Tissue resident memory T cells (T_RM_) have been identified in various tissues, however human liver T_RM_ to date remain unidentified. T_RM_ can be recognized by CD69 and/or CD103 expression and may play a role in the pathology of chronic hepatitis B (CHB) and hepatitis C virus infection (CHC). Liver and paired blood mononuclear cells from 17 patients (including 4 CHB and 6 CHC patients) were isolated and CD8+ T cells were comprehensively analysed by flowcytometry, immunohistochemistry and qPCR. The majority of intrahepatic CD8+ T cells expressed CD69, a marker used to identify T_RM_, of which a subset co-expressed CD103. CD69 + CD8+ T cells expressed low levels of *S1PR1* and *KLF2* and a large proportion (>90%) was CXCR6+, resembling liver T_RM_ in mice and liver resident NK cells in human. Cytotoxic proteins were only expressed in a small fraction of liver CD69 + CD8+ T cells in patients without viral hepatitis, however, in livers from CHB patients more CD69 + CD8+ T cells were granzyme B+. In CHC patients, less intrahepatic CD69 + CD8+ T cells were Hobit+ as compared to CHB and control patients. Intrahepatic CD69 + CD8+ T cells likely T_RM_ which have a reduced cytolytic potential. In patients with chronic viral hepatitis T_RM_ have a distinct phenotype.

## Introduction

The liver is an organ with unique immunologic properties. Generally, a tolerant milieu is maintained in the liver to prevent broad immune activation in response to gut-derived antigens^1^. Hepatotropic viruses, such as hepatitis B (HBV) and C virus (HCV), are therefore thought to specifically target the liver for infection. During an infection, upon encounter with their cognate antigen, antigen-specific T cells undergo clonal expansion and subsequently form a memory T cell population^2^. Recently, it became evident that this memory population does not only consist of a recirculating fraction - detectable in the peripheral blood - but also includes an important tissue resident memory T cell (T_RM_) pool, residing in non-lymphoid organs^3^. T_RM_ can be identified by expression of CD69, identifying a broad population of which a subset of cells co-expresses CD103 (integrin alpha E)^3, 4^. T_RM_ reside in human tissues such as lung, skin and gut and have unique functions^3, 5–7^. For example, resident intrahepatic, but not circulatory CD8+ T cells, are the main effectors in an effective immune response against malaria in mice^8^. In viral hepatitis, local bystander T_RM_ may play an important role in the pathology observed in chronic viral infection of the liver^9^.

Whereas previous data has identified a large CD69 + NK cell population in the liver^10^, data on CD8+ T cells with a tissue resident phenotype in the human liver is lacking, as is knowledge on their phenotype in patients with viral hepatitis. The aim of this study was to examine the presence of intrahepatic T_RM_ in control patients without viral hepatitis, as well as the presence of these cells in the liver from patients who are chronically infected with HBV or HCV.

## Results

### Human intrahepatic CD69 + T cells express a tissue resident phenotype

**Figure 1:**
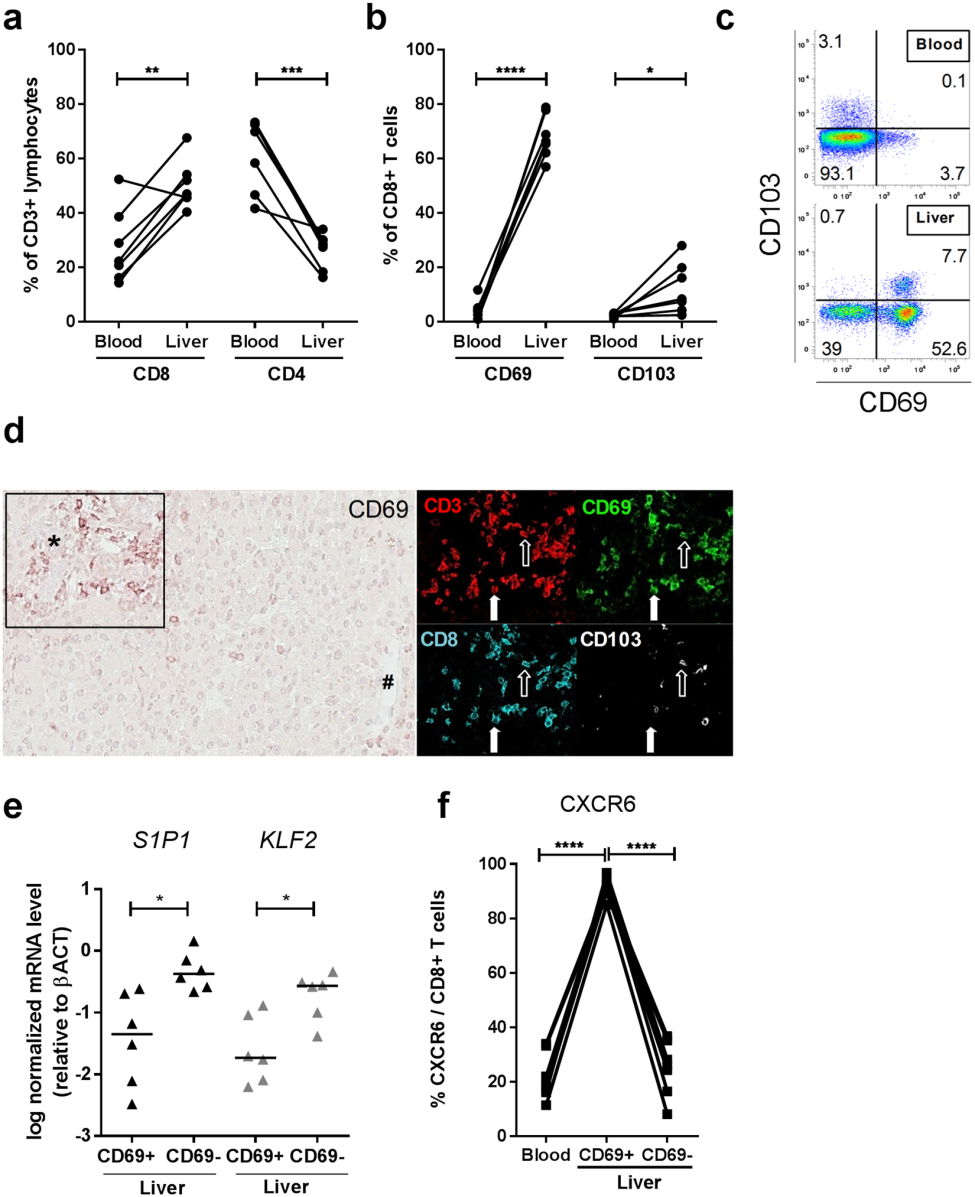
(**a**) Frequency of CD8+ and CD4+ T cells as a percentage of total CD3+ lymphocytes was compared in control blood and liver (n = 7). Statistical analyses; paired t-test. (**b**) Frequency of CD69+ and CD103+ as a percentage of total CD8+ T cells in blood and liver. Statistical analyses; paired t-test. (**c**) Representative flow cytometry plots showing CD69 and CD103 expression, gated on CD8 + CD3+ positive lymphocytes. (**d**) Paraffin embedded formalin-fixed sections of control livers (n = 3)(tumor-free tissue was used) were stained using sequential immunohistochemistry. An immunohistochemical staining of CD69 is shown with a portal field (*) and a central vein (#), and a portal field with co-localization of CD69 + CD8 + CD3+ lymphocytes (filled arrow) and CD69 + CD103 + CD8 + CD3 + lymphocytes (open arrow). Original magnification: 20x. (**e**) Relative expression of *S1PR1* and transcription factor *KLF2* in sorted CD69 + and CD69-CD8+ T cells from the liver (n = 6). Bars indicate median. Statistical analyses; Wilcoxon signed rank test. (**f**) Frequency of CXCR6+ T cells as a percentage of total CD8+ T cells in blood, liver CD69+ and liver CD69-CD8+ T cells. Statistical analyses; paired t-test. *p < 0.05, **p < 0.01, ***p < 0.001, ****p < 0.0001.

In the liver, CD8+ T cells were enriched as compared to the blood (p = 0.0042). Inversely, the liver contained fewer CD4 + T cells than the blood (p = 0.0004, Fig. 1a). To identify intrahepatic T_RM_, we analysed the expression of two markers commonly expressed by T_RM_; CD69 and CD103^5, 11^. In the blood, few CD69 positive cells were present within the CD8+ T cell population (mean 4.3%), while a significant population of CD69 + CD8+ T cells was detected in the liver (mean 68.0%, p < 0.0001, Fig. 1b, gating strategy in Supplementary Fig. 1). In the peripheral blood, mean 2.5% of CD8+ T cells expressed CD103, while mean 12.4% of intrahepatic CD8+ T cells were CD103 positive (p = 0.03, Fig. 1b,c). In contrast to those in the blood, the CD103 + CD8+ T cells detected in the liver co-expressed CD69 (Fig. 1c). Immunohistochemistry revealed localization of CD69 + CD8 + CD3+ positive cells in portal fields, central veins, and parenchymal zones 1–3 (Fig. 1d, Supplementary Fig. 2).

The sphingosine 1-phosphate receptor-1 (S1PR1) is crucial for recirculation of (naïve) T cells^12^, and negatively regulated by CD69^7, 11–13^. *S1PR1* and its regulatory transcription factor Krüppel-like Factor 2 (*KLF2)* were significantly down-regulated in sorted intrahepatic CD69 + CD8+ T cells as compared to CD69-CD8+ T cells (Fig. 1e). Chemokine receptor CXCR6, associated with homing to the liver^10, 14, 15^, was expressed by mean 91.2% of CD69 + CD8+ T cells in the liver, as compared to mean 25.0% of their CD69- liver counterparts (p < 0.0001), and mean 21.9% of peripheral blood CD8+ T cells (Fig. 1f). Additionally, 25–35% of CD69 + CD8+ T cells in the liver were positive for the MR1 tetramer, CD161 and IL-18Rα, specific for mucosa associated invariant T (MAIT) cells (Supplementary Fig. 3)^16^.

### Intrahepatic CD69 + CD8+ T cells are not recently activated

**Figure 2:**
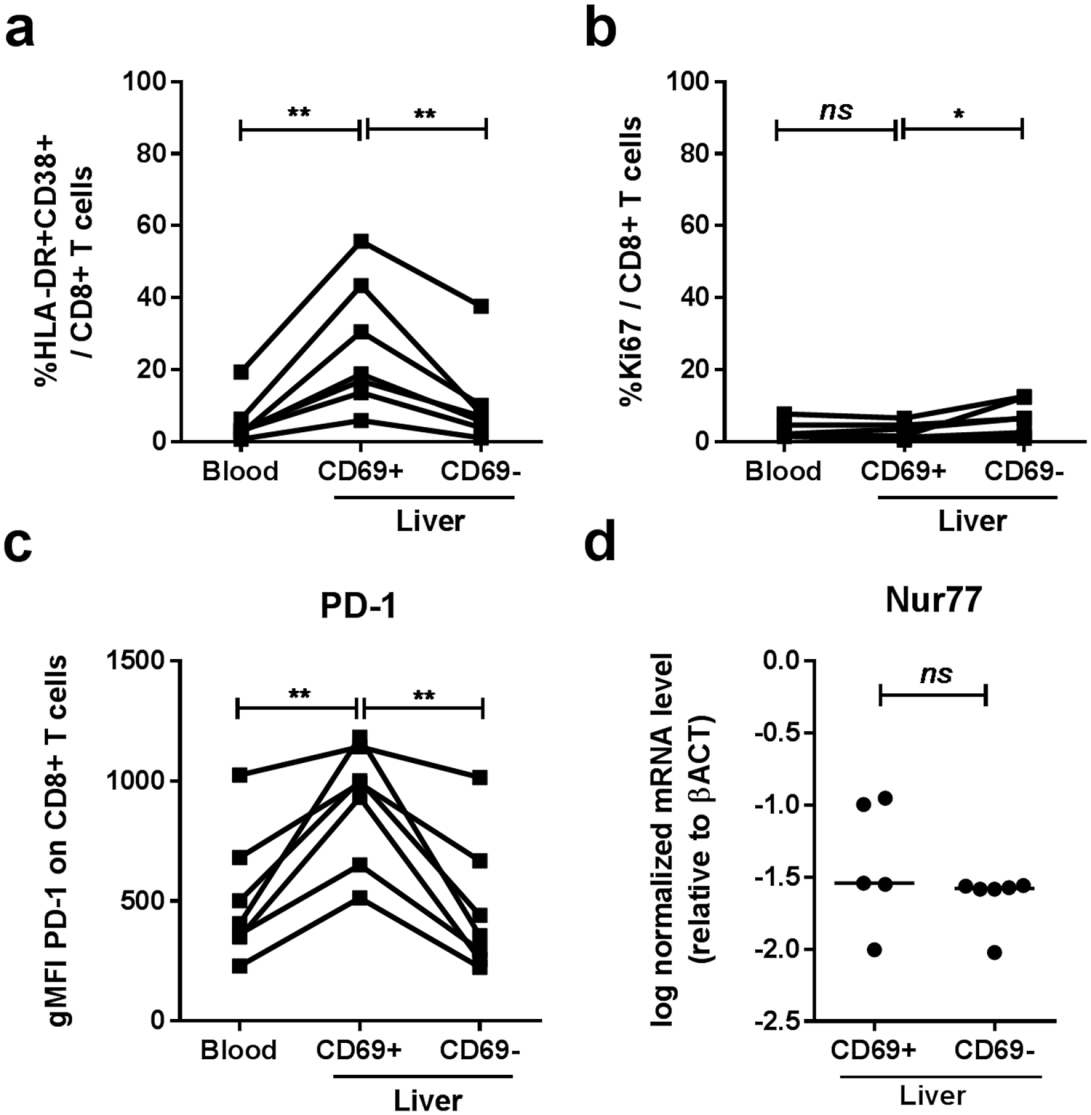
Frequency of HLA-DR + CD38+ double positive (**a**) and Ki67 (**b**) positive cells as a percentage of CD8+ T cells and gMFI of PD-1 (**c**) in control blood vs. liver (CD69+ and CD69-) CD8+ T cells. Statistical analyses; paired t-test. **p < 0.01. (**d**) Relative expression of *Nur77* in sorted CD69+ and CD69-CD8+ T cells from the liver (n = 6, one CD69+ sample was unavailable). Bars indicate median. Statistical analyses; Wilcoxon signed rank test. Abbreviations: ns, non-significant.

In intrahepatic CD69 + CD8+ T cells, the proportion of HLA-DR/CD38 double positive T cells, which in the peripheral blood represent activated cells, was significantly higher (mean 26.4%) than in peripheral blood T cells (mean 5.4%, p = 0.0049, Fig. 2a, Supplementary Fig. 4). Whereas alanine aminotransferase (ALT) levels significantly correlated with the proportion of HLA-DR/CD38 expressing CD8+ T cells in the blood (p = 0.01), in the liver there was no correlation between the two (p = 0.33, Supplementary Fig. 5). The proportion of Ki67 + cells, which represent cells that have recently divided, was significantly higher in intrahepatic CD69-CD8+ T cells as compared to CD69 + CD8+ T cells (mean 6.5% and 1.6% respectively, p = 0.04, Fig. 2b). The proportion of cells expressing apoptosis marker TO-PRO-3, was low in all populations (Supplementary Fig. 6). Programmed death-1 (PD-1), associated with T cell inhibition, was higher expressed on liver CD69 + CD8+ T cells as compared to liver CD69- cells and peripheral blood T cells (Fig. 2c). Other inhibitory molecules were not differentially expressed on intrahepatic CD69 + and CD69- CD8+ T cells (Supplementary Fig. 6). Nur77, an ‘immediate early gene’, up-regulated in response to TCR stimulation^17^, was not differentially expressed in CD69 + and CD69-CD8+ T cells isolated from the liver (Fig. 2d). As expected, the proportion of naïve peripheral blood CD8+ T cells expressing activation markers was low (Supplementary Fig. 7).

### Intrahepatic CD69 + CD8+ T cells are not naïve, but have a memory or effector-memory phenotype, similar to the peripheral blood

**Figure 3:**
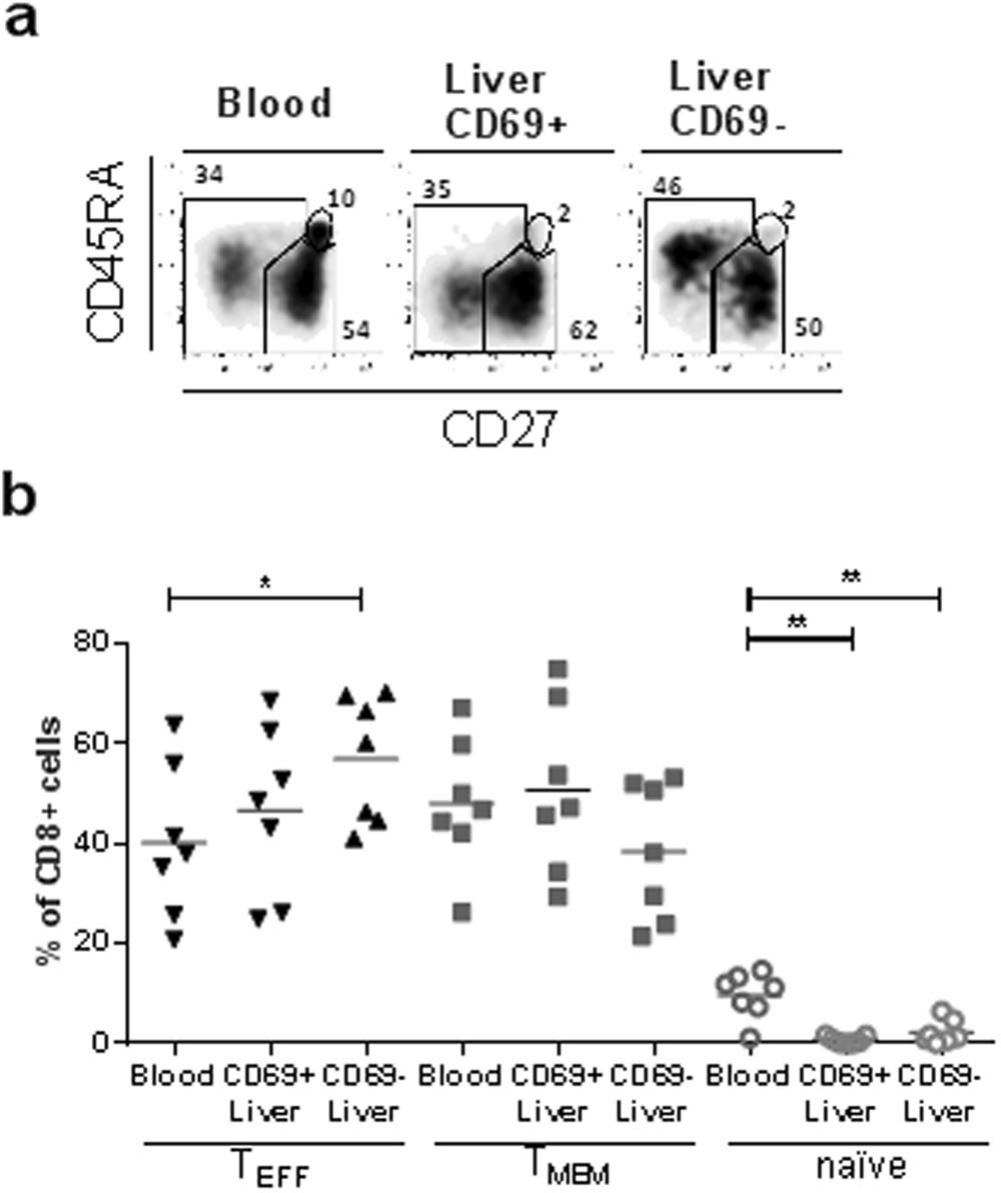
(**a**) Representative flow cytometry plots showing CD27 and CD45RA expression with gates indicating effector memory (T_EFF_, CD45RA ± CD27−), memory (T_MEM_, CD45RA-CD27+) and naïve (CD45RA + CD27 + ) CD8+ T cells in control blood and liver (CD69+ and CD69−). (**b**) Frequency of T_EFF_, T_MEM_ and naïve CD8+ T cells as a percentage of total CD8+ T lymphocytes in control blood and liver (CD69+ and CD69−). Bars indicate mean. Statistical analyses; paired t-test *p < 0.05, **p < 0.01, ****p < 0.0001. Abbreviations: ns, non-significant.

CD8+ T cells can be divided into different subsets, including naïve and memory cells, the latter comprising cells that have been primed by antigen. Antigen experienced cells can be divided into memory T cells (T_MEM_) and effector-type memory T cells (T_EFF_). We identified these populations by CD27 and CD45RA expression, and analysed naïve (CD45RA + CD27+), T_MEM_ (CD45RA-CD27+) and T_EFF_ (CD45RA ± CD27-) T cells (Fig. 3a)^2, 18, 19^. The proportion of T_EFF_ was comparable in blood and intrahepatic CD69 + CD8+ T cells (Fig. 3b). In liver CD69-CD8+ T cells, a significantly higher proportion of CD8+ T cells were T_EFF_ phenotype as compared to the blood (p = 0.01). The proportion of T cells that had a T_MEM_ phenotype was not different between blood, intrahepatic CD69+ and intrahepatic CD69- CD8+ T cells. Naïve cells were significantly less frequent in the CD8+ T cell populations derived from the liver (CD69+ and CD69- mean 0.7%, 2.2% respectively) as compared to the blood (mean 9.7%, Fig. 3a,b).

### The majority of intrahepatic CD69 + CD8+ T_EFF_ cells do not express cytotoxic proteins

**Figure 4:**
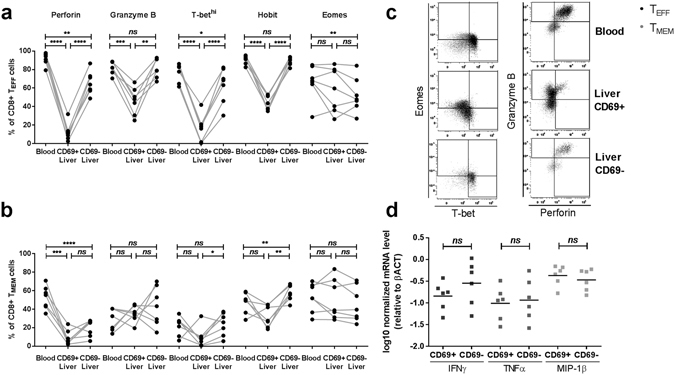
Frequency of perforin+, granzyme B+, Tbet^hi^, Hobit+ and Eomes+ cells as a percentage of CD8+ T_EFF_ (**a**) and T_MEM_ (**b**) in control blood and liver (CD69+ and CD69−). Statistical analyses; paired t-test. (**c**) Representative FACS staining and gating for perforin, granzyme B, Eomes and T-bet in blood and liver CD69 + ad CD69- T_EFF_ and T_MEM_ cells. Statistical analyses; paired t-test. (**d**) Relative expression of cytokines interferon-γ (IFNγ), tumor necrosis factor-α (TNFα) and macrophage inflammatory protein 1β (MIP-1β) in sorted CD69+ and CD69-CD8+ T cells from control livers. Bars indicate median. Statistical analyses; Wilcoxon signed rank test. *p < 0.05, **p < 0.01, ***p < 0.001 ****p < 0.0001. Abbreviations: ns, non-significant.

As intrahepatic NK cells expressing CD69 and CXCR6 have a reduced cytolytic potential^10^, we next analysed the expression of these effector molecules in intrahepatic T_EFF_ and T_MEM_ subsets. The proportion of intrahepatic CD69 + T_EFF_ cells expressing perforin was significantly lower (mean 12.1%) that of blood T_EFF_ cells (mean 91.8%, p < 0.0001) as was the proportion of granzyme B + cells (mean 45.7% and 82.4 respectively, p = 0.0001, Fig. 4a,c). Within the intrahepatic T_EFF_ cell population expressing CD69, significantly less cells expressed cytotoxic proteins as compared to CD69- T_EFF_ cells (p < 0.0001 for perforin and p = 0.001 for granzyme B). In addition, significantly less intrahepatic T_MEM_ expressed perforin (CD69 + ; mean 9.3 and CD69-; mean 19.8%) than in the blood (mean 52.6%, p = 0.0002, Fig. 4b,c), whereas no difference in T_MEM_ granzyme B expression was observed (Fig. 4b,c).

### The majority of CD69 + CD8+ T_EFF_ cells in the liver do not express transcription factors T-bet and Hobit

Memory formation and effector function is tightly regulated by transcription factors such as the T-box transcription factors T-bet, “homolog of Blimp1 in T cells” (Hobit) and Eomesodermin (Eomes)^20–22^. In the liver, a significantly less CD69 + T_EFF_ cells expressed T-bet (mean 16.9%) as compared to blood T_EFF_ (mean 77%, p < 0.0001, Fig. 4a,c). As in the blood, the majority of intrahepatic CD69- T_EFF_ cells expressed T-bet (mean 63%). Hobit was expressed in CD69 + CD8+ T_EFF_ from the liver in significantly less cells (mean 44.2%) than in the blood (mean 90.7%) and CD69- liver CD8+ T cells (mean 88.9%, p < 0.0001). In naïve peripheral blood CD8+ T cells expression these transcription factors was only observed in a small proportion of cells (mean T-bet 1.94%, Hobit 5.7% and Eomes 9.96%, Supplementary Fig. 7).

### Intrahepatic CD69 + T_RM_ express equal amounts of cytokine mRNA as CD69- liver cells

We next investigated the potential of liver-resident T-cells to produce antiviral and proinflammatory cytokines. Cytokine mRNA content was assessed in intrahepatic CD69+ and CD69- cells. No differences were observed in interferon-γ (IFN-γ), tumor necrosis factor-α (TNF-α) and macrophage inflammatory protein-1β (MIP-1β) mRNA expression between CD69+ and CD69-CD8+ T cells (Fig. 4d).

### Intrahepatic CD69 + CD8+ T cells express a distinct phenotype in chronic viral hepatitis

**Figure 5:**
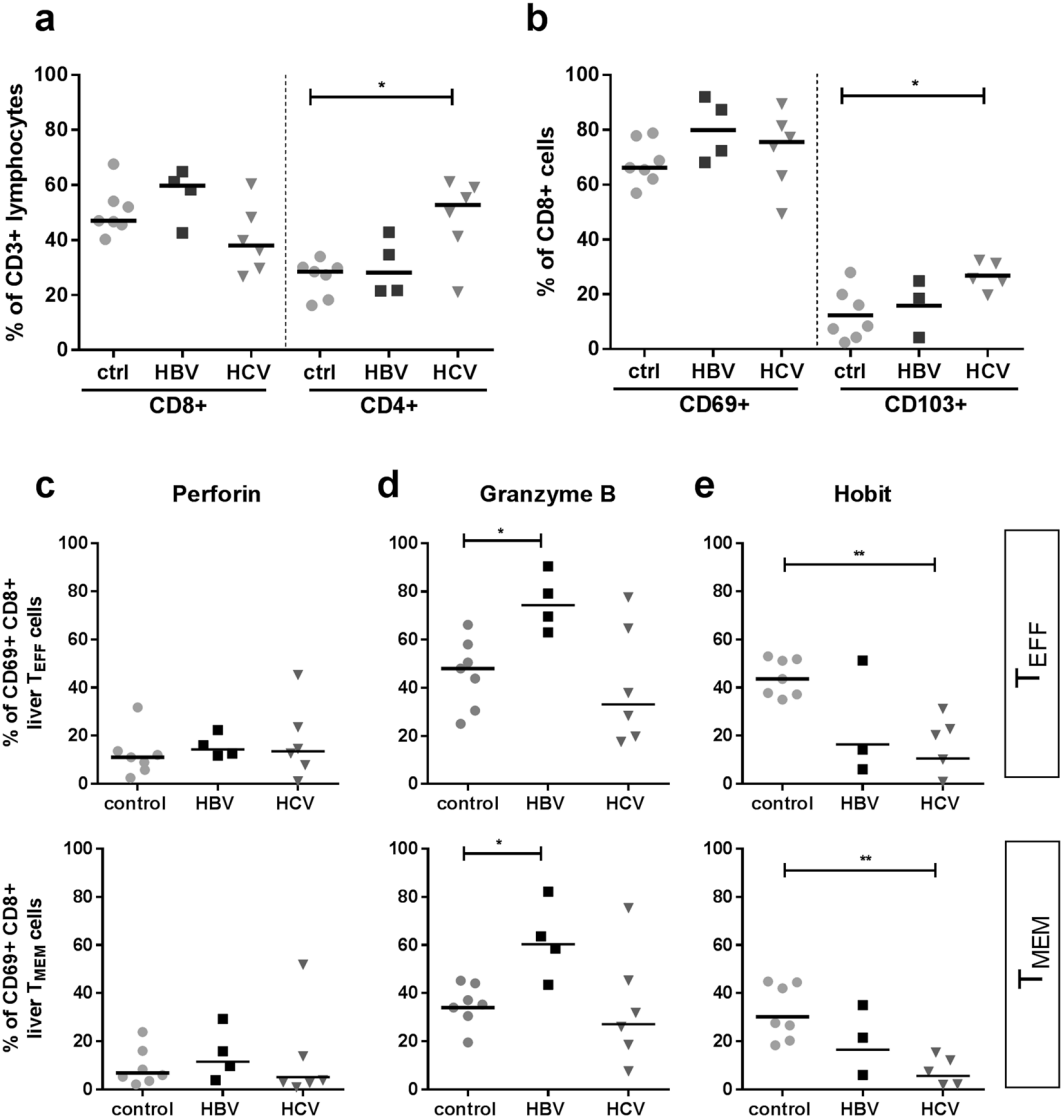
(**a**) Frequency of CD8+ and CD4+ T cells as a percentage of total CD3+ T lymphocytes in control (ctrl, n = 7), CHB infected (HBV, n = 3–4) and CHC infected (HCV, n = 5–6) livers (number of samples depending on sample availability). Bars indicate median. Statistical analyses; Mann-whitney test. (**b**) Frequency of CD69+ and CD103+ as a percentage of total CD8+ T lymphocytes in control, HBV and HCV livers. Bars indicate median. Statistical analyses; Mann-whitney test. Frequency of perforin+ (**c**), granzyme B+ (**d**) and Hobit+ (**e**) CD8+ T cells as a percentage of intrahepatic CD69 + CD8+ T_EFF_ (upper graphs) and T_MEM_ (lower graphs). Bars indicate median. Statistical analyses; Mann-whitney test. *p < 0.05, **p < 0.01.

The proportion of CD8+ T cells in the liver of patients with HBV and HCV was similar to that of control patients (Fig. 5a). The proportion of CD4 + T cells was significantly higher in the livers of patients with CHC (median 52.8%) as compared to control patients (median 28.5%, p = 0.02, Fig. 5a). As in control patients, the majority of CD8+ T cells in the liver of patients with CHB or CHC expressed CD69 (median 66.2%, 79.9% and 75.6%). A minority of these cells co-expressed CD103 and this population was significantly expanded in patients with CHC (median 25.7%) as compared to control patients (median 8.4%, p = 0.03, Fig. 5b). Similar to control patients, in CHB and CHC patients, most intrahepatic CD69 + CD8+ T cells were CXCR6 positive (Supplementary Fig. 8a). HLA-DR/CD38, Ki67 and PD-1 were similarly expressed on CD69 + CD8+ T cells in CHB, CHC and control patients. In CHB patients, significantly more CD69 + CD8+ T_EFF_ cells expressed cytotoxic protein granzyme B (median 74.4%) as compared to controls (median 48.1%, p = 0.012, Fig. 5d), while this was similar between CHC patients and controls. In CHC patients, significantly less CD69 + CD8+ liver T cells expressed Hobit as compared to control CD69 + CD8+ T cells (T_EFF_ as well as T_MEM_, *p* = 0.0025, Fig. 5e). These differences between patient groups were not observed in intrahepatic CD8+ T cells that were CD69 negative (data not shown). The percentage of cells expressing perforin and transcription factors T-bet and Eomes did not differ in patients with CHB and CHC and controls (Fig. 5c, Supplementary Fig. 8b).

## Discussion

In this study we have for the first time in man characterized a population of CD69 + CD8+ T cells with a tissue resident-like phenotype which is abundantly present in the liver, but not in the peripheral blood. Expression of CD69 identifies T_RM_ in many different human tissues^3, 7^, as well as on the majority of intrahepatic T_RM_ in mice and resident-like NK cells in the human liver^10, 13, 23^. A small proportion of intrahepatic CD69 + CD8+ T cells expressed CD103, in accordance with data on mouse liver T_RM_^13^. Subsets of human T_RM_ including those in lung, brain, skin and gut^3, 24^ co-express CD103, which forms a heterodimer with integrin β7 and recognizes E-cadherin, which is expressed on hepatocytes and bile duct epithelium. In patients with CHC, this population was enriched, possibly causing portal infiltrates and liver pathology^25^. CD69 is known to antagonize the expression of the S1P receptor S1PR1^12^, which can detect an S1P gradient leading out of the tissues^11, 12, 26^. Lower expression of *S1PR1* and its regulatory transcription factor *KLF2* in intrahepatic CD69 + CD8+ T cells suggests that these cells are not able to egress from the liver, and are indeed T_RM_, as described for mouse liver T_RM_^27^. Parabiosis experiments, in which mice share their circulation, revealed that nearly all CD8+ T cells expressing CD69 are non-circulatory and interestingly, these T_RM_ could be localized in the parenchyma as well as within large vessels in the liver^23^. Here, immunohistochemistry localized CD69 + CD8+ T cells in the parenchyma as well as within portal fields of the liver. Expression of CXCR6 has been associated with human liver infiltrating lymphocytes^14^, intrahepatic NK cells^10^, and protective responses against malaria in mice^15^. High expression of CXCR6 could facilitate maintenance in the liver as its ligand, CXCL16, is present on hepatocytes and liver sinusoidal endothelial cells (LSEC) in healthy and HCV infected livers^14, 28^. Few naïve cells could be identified in the population of CD69 + CD8+ T cells from the liver, supporting the hypothesis that CD69 identifies a distinct non-circulating population of memory T cells^29, 30^.

CD69 has originally been identified as a marker of recent activation. To investigate the activation state of CD69 + CD8+ T cells, we examined the expression of other markers of recent and chronic stimulation. Low numbers of blood and liver CD8+ T cells were Ki67 positive, suggesting the absence of persistent T cell activation as observed in malaria specific intrahepatic T_RM_^27^. Nur77, a factor upregulated after recent TCR activation^17^, was not differentially expressed in CD69+ and CD69-CD8+ T cells from the liver, suggesting that the expression of CD69 was not promoted by recent TCR cross-linking. CD69 + CD8+ T cells in the liver contained a higher fraction of HLA-DR/CD38 double positive cells, which is consistent with data regarding lung residing CD8+ T cells^31^. The proportion of HLA-DR expressing cells correlated with ALT in the peripheral blood but not in the liver, suggesting in the liver immune activation is not the primary mechanism of HLA-DR/CD38 upregulation. In addition, CD69 + CD8+ T cells expressed PD-1 at higher levels than CD69- liver CD8+ T cells and blood CD8+ T cells which could play a role in maintaining local tolerance, similar to what has been reported for CD103 + T_RM_ in the brain^32^. Local induction of apoptosis to induce tolerance did not seem to play a role in the liver^33^.

A hallmark of effector type CD8+ T cells in the periphery is the presence of intracellular cytotoxic proteins, such as perforin and granzyme B^2^. Significantly less CD69 + CD8+ T_EFF_ cells in the liver expressed cytotoxic proteins, as compared to peripheral effector cells, similar to what has been observed in T_RM_ in the skin^34^, brain^35^, and lung^36^, and in intrahepatic NK cells^10^. A reduced cytolytic activity in the brain has been postulated to prevent immune pathology in response to low or non-specific stimuli^35^, a similar mechanism could be at play in the liver. On the other hand, as part of the intrahepatic CD69 + CD8+ T cell population consisted of MAIT cells, it cannot be excluded that some of these differences could be caused by this population which is generally low in cytotoxic content^37^. In CHB patients, more liver-resident T_EFF_ as well as T_MEM_ cells expressing granzyme B were observed than in CHC and control patients, possibly antagonizing tolerance and adding to liver damage. The infiltration of a dense non-virus specific T cell population has previously been suggested to drive liver damage in viral hepatitis^9, 38^.

T-bet, Hobit and Eomes are the leading transcription factors thought to govern the process of T cell differentiation^21, 22, 39–41^, as well as T_RM_ development^13, 22^. T-bet and Hobit were expressed by significantly less cells in the intrahepatic CD69 + CD8+ T cell population as compared to peripheral blood CD8+ T cells. As these transcription factors play an important role in the regulation of cytolytic protein expression^41, 42^, the distinct transcriptional profile observed here possibly induced a reduction in perforin and granzyme B expression. A significantly larger proportion of intrahepatic CD69- CD8+ cells expressed T-bet and Hobit as compared to CD69+ cells, whereas the IFN-γ mRNA content did not differ between these populations. This suggests that in the liver, mechanisms additional to T-bet and Hobit are regulators of IFN-γ production^21, 41, 43^. Hobit expression has been shown to be important in the development of liver T_RM_ in mice^13^. As Hobit expression was observed in liver CD69+ T cells, this transcription factor may also be involved in T_RM_ development in humans. However, significantly less intrahepatic CD69 + CD8+ T cells expressed Hobit as compared to intrahepatic CD69- T cells and blood CD8+ T_EFF_ cells, which are well known to express Hobit^21^. Furthermore, in CHC livers, the proportion of Hobit expressing cells was reduced, which indicates that an alternative differentiation process occurs in these cells.

Previous data on human intrahepatic NK cells did not show differences between healthy donor tissue obtained from organ donors and resection margins of liver metastases^10^. However, it would be interesting to evaluate the phenotype of liver the T_RM_ population in truly healthy subjects in further studies. Other groups have already identified the presence of substantial populations of T_RM_ in many tissues in organ donors^3, 7^. Similar to their results, in this study we have identified an intrahepatic T cell population with a specific tissue resident phenotype. The presence of a chronic viral infection in the liver seems to infer specific changes to the differentiation status of this intrahepatic CD69 + CD8+ T cell population, and suggests an altered cytotoxic potential in CHB and specific regulation of differentiation and homing capabilities in CHC infection. Understanding the role of tissue-resident T cells in chronic viral hepatitis may help the development of future therapeutic interventions enabling the prevention of liver cirrhosis and hepatocellular carcinoma.

## Patients and Methods

### Patient samples

We included 17 patients of which 7 did not have viral hepatitis (controls), 4 had chronic HBV (CHB) infection and 6 had chronic HCV (CHC) infection. Patient characteristics are listed in Table 1. The study was approved by the Ethical Review Board of the Academic Medical Center, Amsterdam, The Netherlands and all patients gave written informed consent in accordance with the Declaration of Helsinki. The study was performed in accordance with Good Clinical Practice guidelines, Good Laboratory Practice guidelines and local regulatory requirements. Liver tissue with paired blood samples were obtained from patients undergoing surgical liver resection (indications listed in Table 1). From the resected liver tissue, the non-affected, tumor-free margin surrounding the pathology was obtained. Liver samples were perfused to remove any residual blood, disrupted and treated with collagenase IV (Worthington Biochemical Corporation, CA, USA) and DNAse I (Sigma-Aldrich, MO, USA). Mononuclear cells from blood and liver were isolated using standard density-gradient centrifugation and cryopreserved for later analysis.

**Table 1:** Patient characteristics.

Case#	Viral status	Gender	Age	Reason surgery	Cirrhosis*	Genotype	ALT (U/l)
1	Control	m	81	cholangiocarcinoma	no		42
2	Control	m	81	liver metastasis CRC	no		30
3	Control	f	50	liver metastasis CRC	no		33
4	Control	m	68	HCC	no		24
5	Control	f	37	multiple adenomas	no		50
6	Control	m	68	intrahepatic gallstones	no		32
7	Control	m	67	liver metastasis CRC	no		18
8	CHB	m	51	HCC	yes	C	81
9	CHB	m	57	HCC	F3/F4	C	33
10	CHB	m	47	cholangiocarcinoma	F1	unknown	43
11	CHB	m	64	HCC	F3	unknown	11
12	CHC (HBVc)	m	58	HCC	yes	1b	57
13	CHC (HBVc)	m	58	HCC	F2/F3	1b	129
14	CHC	m	59	HCC	yes	1a	154
15	CHC	m	61	HCC	yes	1a	111
16	CHC	m	52	HCC	yes	3a	30
17	CHC	m	55	HCC	yes	4	112

*Cirrhosis based on METAVIR score at histology. Abbreviations; ALT, alanine aminotransferase; m, male; f, female; CRC, colorectal carcinoma; HCC, hepatocellular carcinoma; CHB, chronic hepatitis B; CHC, chronic hepatitis C; HBVc, cleared acute hepatitis B virus.

### Flow cytometry

Cells were thawed and stained with different combinations of fluorescent label-conjugated mouse monoclonal antibodies (listed in the Supplementary Materials). For intracellular staining, cells were fixed after surface staining, permeabilized, and stained with fluorescent label-conjugated mouse monoclonal antibodies (Supplementary Materials). All measurements were done on an LSR Fortessa flowcytometer (BD Biosciences, CA, USA) and analyzed by FlowJoMacV 9.7.5 software (Tree Star Inc., OR, USA).

### Immunohistochemistry

Paraffin embedded formalin-fixed sections (4 μm) of non-affected liver tissue from control patients (n = 3) were stained using sequential immunohistochemistry^44^. Sections were deparaffinized and rehydrated in up to 96% ethanol. Endogenous peroxidase activity was blocked by incubation with 0.3% H2O2 in methanol. Antigen retrieval was performed at 120 °C under pressure in a Tris/EDTA buffer. The sections were stained overnight with anti-CD69 (mouse IgG1, clone CH11, Abcam, Cambridge, UK), followed by incubation with polyHRP-anti-mouse IgG (ImmunoLogic, Duiven, Netherlands). HRP activity was demonstrated with VectorNovaRed (Vector Laboratories, CA, USA). The slides were counterstained with hematoxilin, coverslipped and scanned in a Philips Ultra Fast Scanner 1.6RA (Philips, Eindhoven, Netherlands). After removal of the coverslip, the sections were stripped in a Tris-SDS buffer at 50 °C. Next, they were incubated with anti-CD103 (rabbit IgG, clone EPR4166, Abcam, Cambridge, UK) and subsequent polyHRP-anti-rabbit IgG (ImmunoLogic, Duiven, Netherlands). HRP activity was demonstrated as above. This procedure was repeated with anti-CD8 (mouse IgG1, clone C3/144B, Dako, Glostrup, Denmark) and anti-CD3 (rabbit IgG1, clone Sp7, Thermo scientific, NH, USA). Images were analysed with Fiji Image J software^45^. Pseudocolors were applied to provide co-localization images.

### Cell sorting and RNA extraction

Liver lymphocytes from 6 control patients were sorted using fluorescent label-conjugated mouse monoclonal antibodies for CD3, CD8 and CD69 (Supplementary Materials). Liver CD3 + CD8+ lymphocytes were sorted into a CD69+ and CD69- fraction on a Sony SH800 cell sorter (Sony biotechnology Inc., CA, USA). After sorting, RNA was extracted with the AllPrep isolation kit (Qiagen, CA, USA).

### Reverse transcription quantitative polymerase chain reaction (RT-qPCR)

Reverse transcription of mRNA was performed using M-MLV reverse transcriptase and oligo-dT primers. Relative quantification of gene expression was determined with the LightCycler 2.0 or LightCycler 480 Real-Time PCR System (Roche Applied Science, CT, USA*)* using the SYBR Green PCR Master Mix and gene specific primers (Supplementary Table 1). mRNA expression levels were normalized to the arithmetic mean of the housekeeping gene *ACTB* using the comparative Ct method, and log10 transformed for analysis.

### Data availability

The datasets generated during and/or analysed during the current study are available from the corresponding author on reasonable request.

### Statistical analyses

Statistical analyses were performed using GraphPad Prism 6 software (GraphPad Software Inc., CA, USA). For paired data, the paired t-test (Gaussion distribution) or Wilcoxon signed rank test (non-Gaussion distribution) was used. Similarly, for non-paired data, the t-test or Mann–Whitney U test was used. Correlations were calculated using the Spearman rank test. Two sided p-values below 0.05 were considered to be statistically significant.

## Electronic supplementary material

Supplementary data
