# Supplementary material for: Human intrahepatic CD69 + CD8+ T cells have a tissue resident memory T cell phenotype with reduced cytolytic capacity
Source: Sci Rep. 2017 Jul 21;7:6172. doi: 10.1038/s41598-017-06352-3 (PMC5522381; doi:10.1038/s41598-017-06352-3)
Supplement: Supplementary file 1 — Supplementary data [file 41598_2017_6352_MOESM1_ESM.pdf]

Supplementary Material

Human intrahepatic CD69+CD8+ T cells have a tissue resident memory T cell phenotype with reduced cytolytic capacity

Femke Stelma, Annikki de Niet, Marjan J. Sinnige, Karel A. van Dort, Klaas P.J.M. van Gisbergen, Joanne Verheij, Ester M.M. van Leeuwen, Neeltje A. Kootstra‡ and Hendrik W. Reesink ‡*

Supplementary Methods

Fluorescent label-conjugated monoclonal antibodies used for FACS phenotyping.

CD3-V500, CD14-PE-CF594, CD19-PE-CF594, CD27-BUV737, CD127-BV421, CD161-APC, HLA-DR-FITC, CD38-Pe-Cy7, CD69-Pe-Cy7, Perforin-FITC, Ki67-Pe-Cy7, CTLA-4-BV785, TIM-3-PE-CF594 (BD Biosciences, CA, USA), CD4-PerCP eFluor710, CD8-AF700, CD45RA-eFluor605NC, PD1-PE, Eomes-PerCP eFluor710, Tbet-Pe-Cy7, IL-18Rα-FITC (eBioscience, CA, USA), CD8-BV785, CXCR6-BV421, PD-1-PerCP-Cy5.5, 2B4-PE, LAG-3-PE-Cy7 (Biolegend, CA, USA), CD69-APC and Red-fluorescent reactive dye for live/dead staining (Invitrogen, OR, USA), CX3CR1-PE (MBL International, Naka-ku Nagoya, Japan), GranzymeB-PE, Hobit-IgM and αIgM-PE (Sanquin, Amsterdam, The Netherlands), CD103-FITC (DAKO, Glostrup, Denmark),

Tetramer: MR1-BV421 (National Institutes of Health tetramer core facility, Atlanta, USA)

Fluorescent label-conjugated monoclonal antibodies used for sorting.

CD3-PE (eBioscience, CA, USA), CD8-BV421 (BD Biosciences, CA, USA), CD69-APC (Invitrogen, OR, USA).

Methods for qPCR

The qPCR program consisted of 10 minutes at 95°C, followed by 50 cycles (10 sec at 95°C, 20 sec at 58°C, 30 sec at 72°C) finishing with a melting curve (5 sec at 95°C, 1 min at 65°C,

continuous melting to 97°C, and 10 sec at 40°C). For Nur77 a different program was used: 2 min at 50°C, 2 min at 95°C, followed by 55 cycles (5 sec at 94°C, 15 sec at 55°C, 15 sec at 72°C) finishing with a melting curve. PCR product specificity was assessed by melting curve analysis.

Gene	Primer	Sequence (5' to 3')
ACTB	F	GGGTCAGAAGGATTCCTATG
	R	GGTCTCAAACATGATCTGGG
IFNG	F	TGAATGTCCAACGCAAAGCA
	R	CTGTTTTAGCTGCTGGCGAC
TNFA	F	GGCGTGGAGCTGAGAGATA
	R	CAGCCTTGGCCTTGAAGA
MIP1B/CCL4	F	GCTTCCTCGCAACTTTGTGG
	R	TCACTGGGATCAGCACAGAC
PRF1	F	CACCCTGACTTCAAGAGGGC
	R	GGATGAAGTGGGTGCCGTAG
GZMB	F	TGCGAATCTGACTTACGCCAT
	R	GGAGGCATGCCATTGTTTCG
NUR77	F	AGTGCAGAAAAACGCCAAGT
	R	TTCGGACAACTTCCTTCACC

Supplementary table 1 Primers used for qPCR.

Supplementary Figures

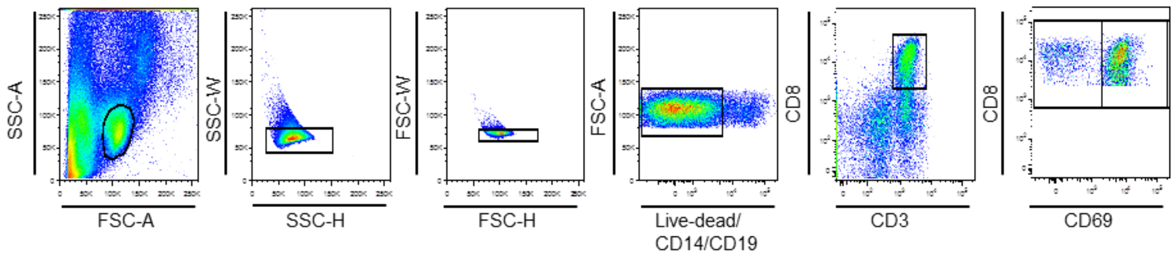

Supplementary figure 1. Gating of liver CD69+ and CD69- CD8+ T cells.

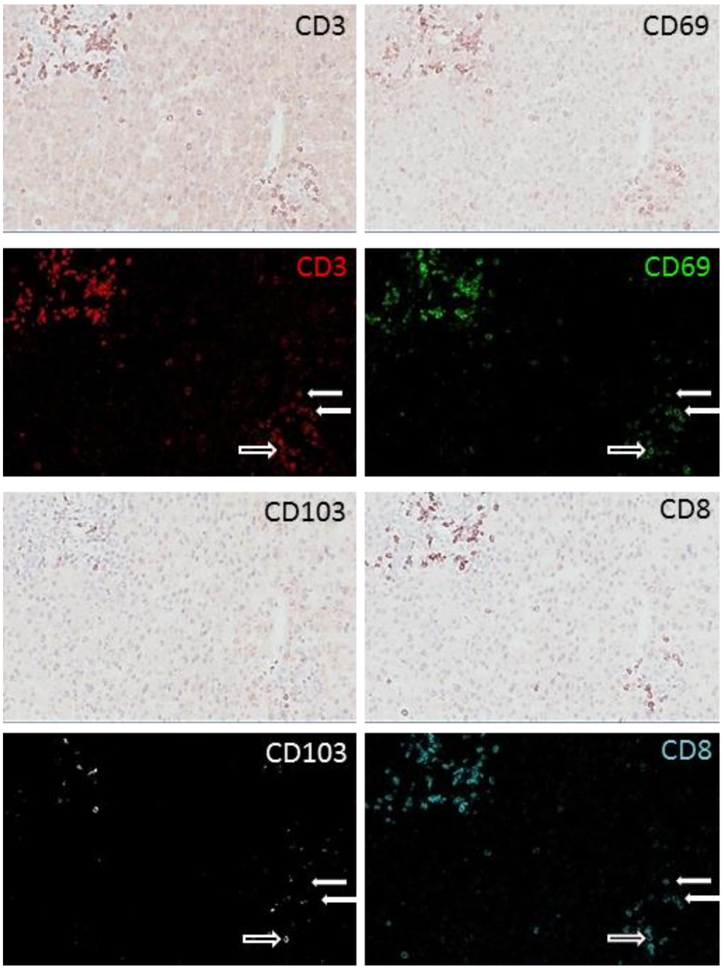

Supplementary figure 2. Immunohistochemical stainings before and after processing in ImageJ.

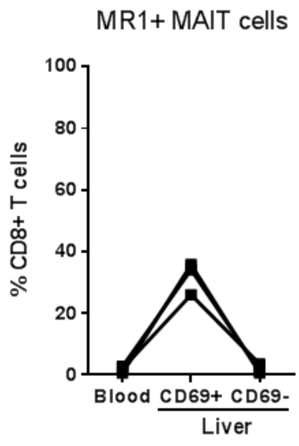

Supplementary figure 3. MR1+CD161IL-18Rα+ mucosa associated invariant T (MAIT) cells.

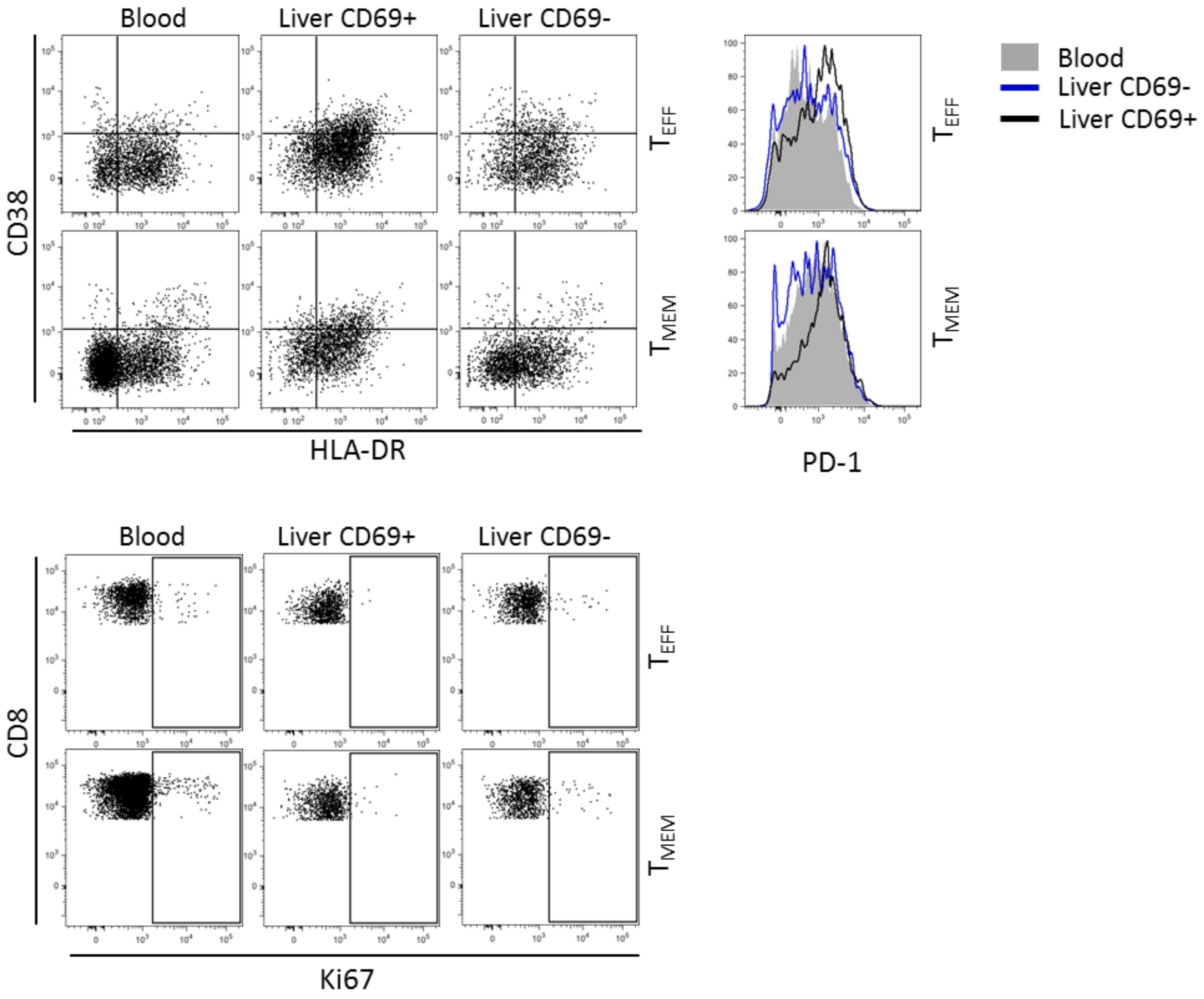

Supplementary figure 4. Representative staining for various surface markers on T_{EM} and T_{MEM} in blood and liver CD69+/CD69- CD8+ T cells.

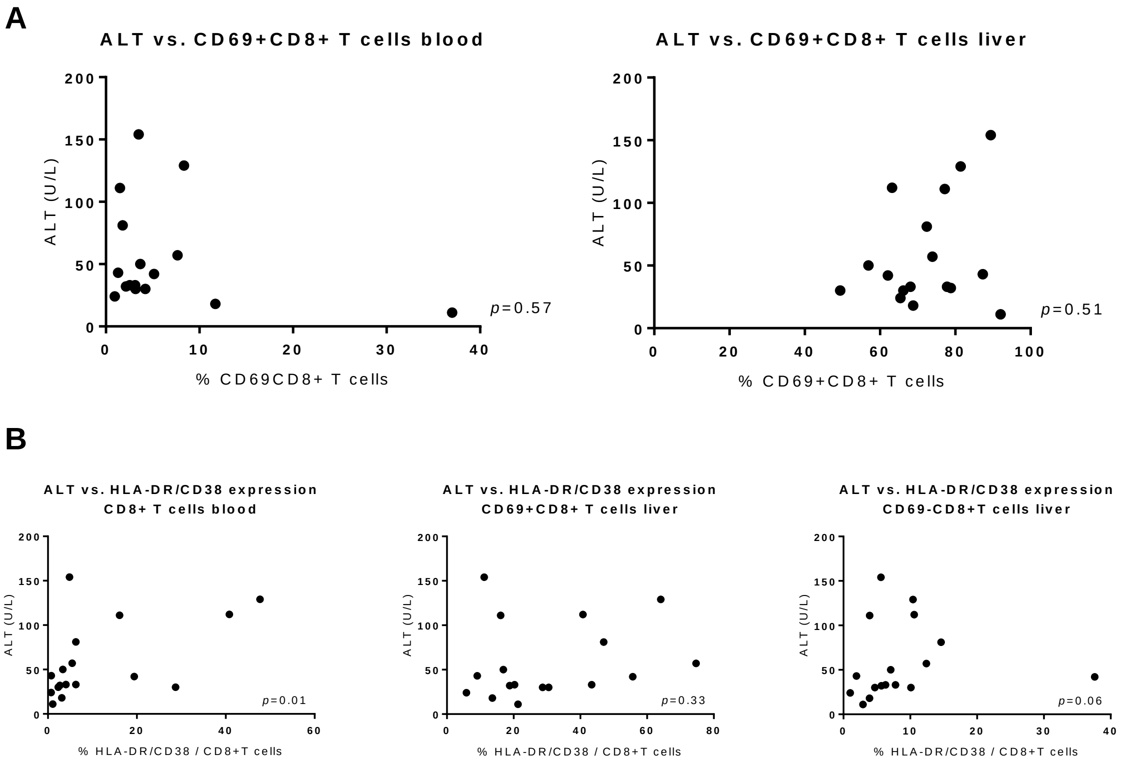

Supplementary figure 5. (A) Correlation between ALT levels and the proportion of CD69 positive CD8+ T cells in the blood and liver. (B) Correlation between ALT levels and (left) the proportion of CD8+ T cells expressing HLA-DR/CD38 in the blood, (middle) the proportion of CD69+CD8+ T cells expressing HLA-DR/CD38 in the liver, and (right) the proportion of CD69-CD8+ T cells expressing HLA-DR/CD38 in the liver.

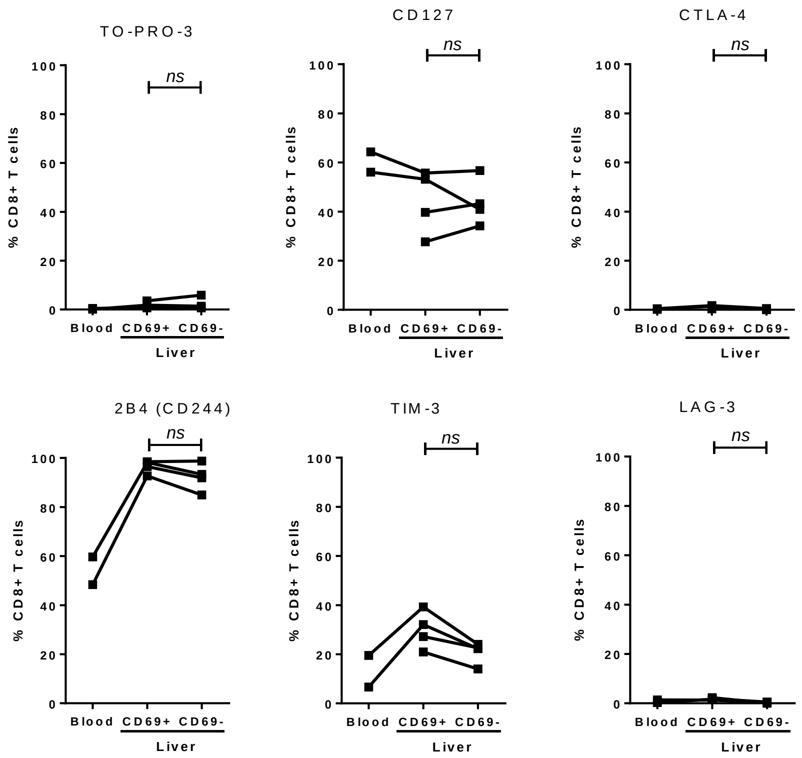

Supplementary figure 6. Proportion of TO-PRO-3, CD127, CTLA-4, 2B4, TIM-3 and LAG-3 positive CD8+ T cells in blood and intrahepatic C69+ and CD69- cells. Four livers samples and 2 paired PBMC samples were available for measurements. Statistical analyses; Wilcoxon signed rank test.

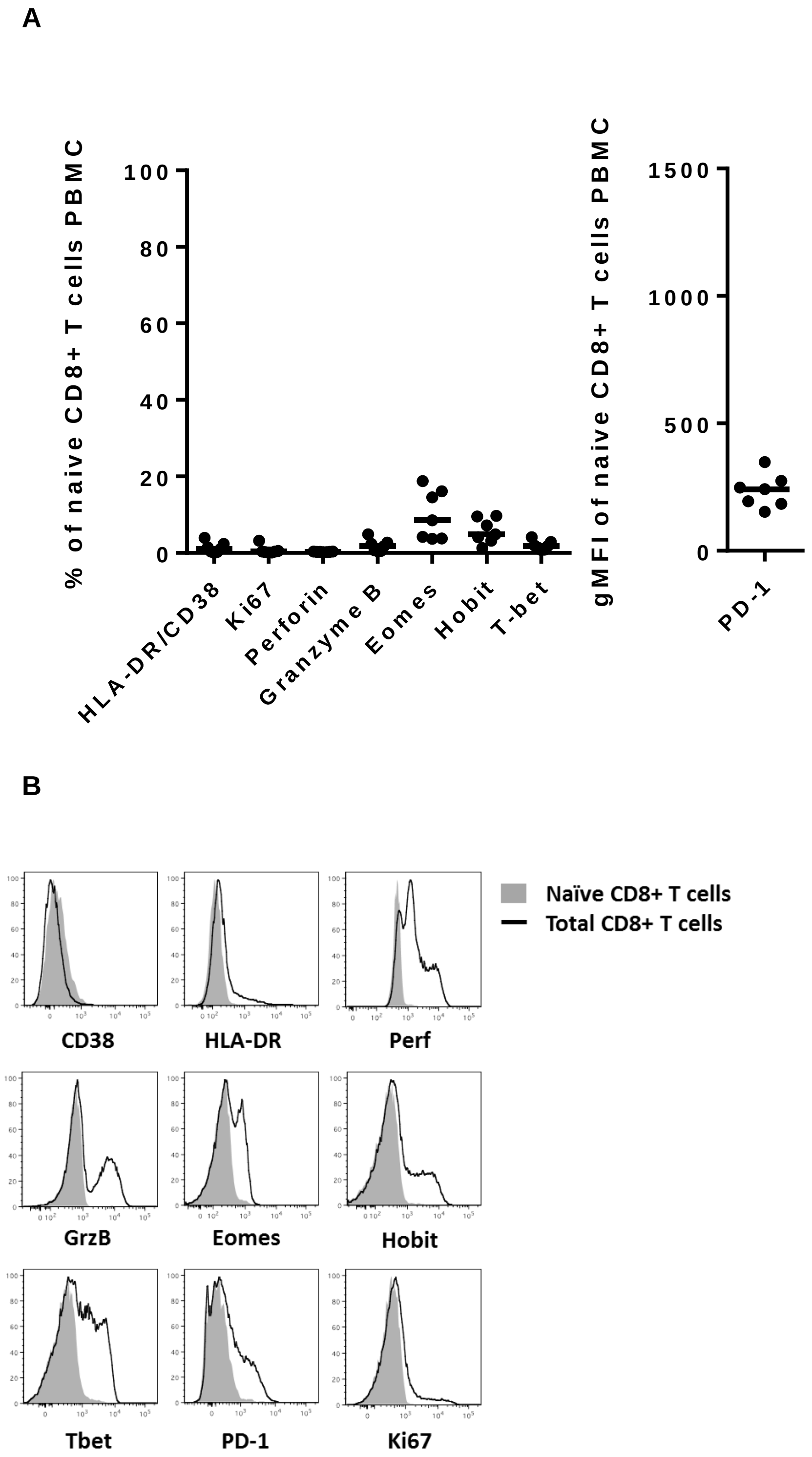

Supplementary figure 7. (A) Proportion of naïve CD8+ T cells in the peripheral blood expressing HLA-DR/CD38, Ki67, Perforin, Granzyme B, Eomes, Hobit, T-Bet. Expression level of PD-1 on naïve CD8+ T cells in the peripheral blood. (B) Expression of several markers on naïve CD8+ T cells, compared to the total CD8+ T cell population (peripheral blood).

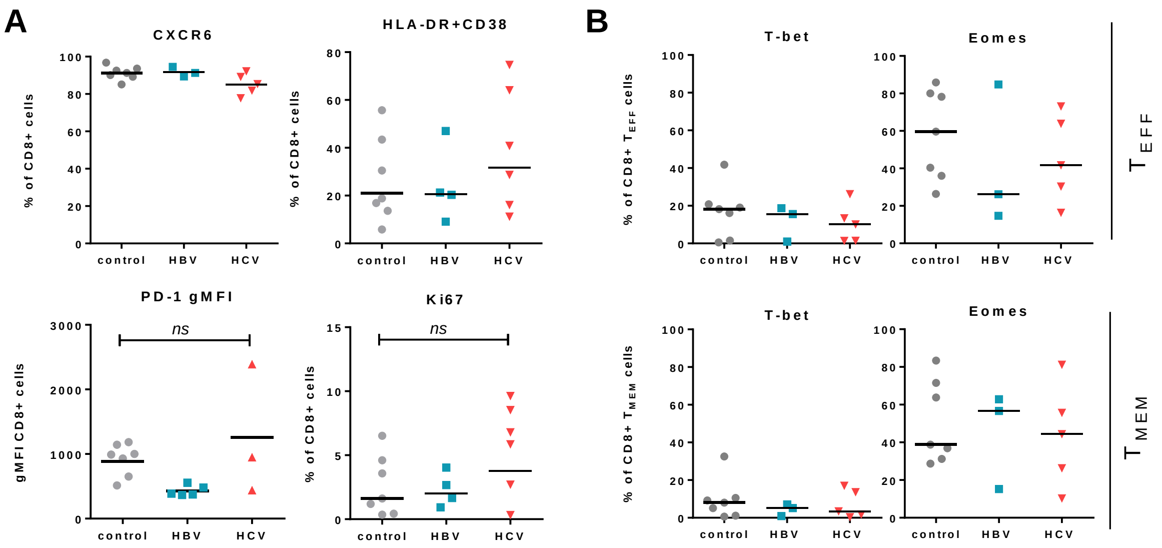

Supplementary figure 8. Liver T_{RM} (CD69+) cells were analysed in 7 control patients, 4 patients with CHB and 6 patients with CHC. Proportion of CD69+CD8+ T cells expressing various markers.
